# *In situ *detection of non-polyadenylated RNA molecules using Turtle Probes and target primed rolling circle PRINS

**DOI:** 10.1186/1472-6750-7-69

**Published:** 2007-10-18

**Authors:** Magnus Stougaard, Jakob S Lohmann, Magdalena Zajac, Stephen Hamilton-Dutoit, Jørn Koch

**Affiliations:** 1 Institute of Pathology, Aarhus University Hospital, Nørrebrogade 44 building 18A, DK-8000 Aarhus C, Denmark

## Abstract

**Background:**

In situ detection is traditionally performed with long labeled probes often followed by a signal amplification step to enhance the labeling. Whilst short probes have several advantages over long probes (e.g. higher resolution and specificity) they carry fewer labels per molecule and therefore require higher amplification for detection. Furthermore, short probes relying only on hybridization for specificity can result in non-specific signals appearing anywhere the probe attaches to the target specimen. One way to obtain high amplification whilst minimizing the risk of false positivity is to use small circular probes (e.g. Padlock Probes) in combination with target primed rolling circle DNA synthesis. This has previously been used for DNA detection *in situ*, but not until now for RNA targets.

**Results:**

We present here a proof of principle investigation of a novel rolling circle technology for the detection of non-polyadenylated RNA molecules *in situ*, including a new probe format (the Turtle Probe) and optimized procedures for its use on formalin fixed paraffin embedded tissue sections and in solid support format applications.

**Conclusion:**

The method presented combines the high discriminatory power of short oligonucleotide probes with the impressive amplification power and selectivity of the rolling circle reaction, providing excellent signal to noise ratios in combination with exact target localization due to the target primed reaction. Furthermore, the procedure is easily multiplexed, allowing visualization of several different RNAs.

## Background

DNA and RNA molecules in situ have traditionally been studied by in situ hybridization (ISH). ISH originally utilized probes in the form of radioactively labeled rRNA, visualized by autoradiography [1,2]. Subsequently, various non-isotopic probe labels have also been used, usually detected with immunoenzymatic methods [3] or fluorescent *in situ *hybridization (FISH) [4,5]. In order to generate sufficient signal, non-isotopic ISH methods usually use long probes or multiple probe cocktails for binding of sufficient number of label molecules to each target. These probes or probe cocktails are in most cases combined with some form of signal amplification such as tyramide signal amplification (TSA), a technique that can increase FISH signal intensity 10–20 fold [6]. However, long probes pose a problem since affinity and specificity for nucleic acid probes usually are inversely correlated, meaning that whilst a probe's affinity for a target increases so does the risk of non-specific binding [7]. Long probes are also not well suited for the discrimination of minor sequence variations. Artificial nucleic acids, such as PNA- and LNA-oligonucleotides, have been utilized as probes, allowing higher hybridization temperatures and increased specificity of the ISH-probes [8,9]. Short horseradish peroxidase (HRP) conjugated oligonucleotides have been used for detection of RNA, using TSA and fluorescently labeled antibodies [10]. Traditional ISH-methods often rely strictly on hybridization and stringent washing for specificity. Therefore, background staining will increase along with the specific signals as result of non-specific binding of the probe. This limits detection of rare targets [11]. Furthermore most amplification techniques used, such as TSA, are not well suited for multiplexing and since both specific and non-specific signals are amplified careful optimization of each hybridization event is required [12].

Another method used for in situ detection of nucleic acids is the primed *in situ *labeling (PRINS) technique. The PRINS technique is based on the generation of detectable DNA by performing a DNA polymerization *in situ*. Traditionally this has been done by using short synthetic oligonucleotides which are hybridized to a target nucleic acid, and used as primers in the subsequent DNA polymerization step during which hapten- or fluorescent-labeled nucleotides are incorporated for tagging sites of DNA synthesis [13]. PRINS has usually been performed on repetitive DNA sequences [13,14], although it has been shown to allow both single copy gene detection [15], and mRNA detection [16].

Thus, existing *in situ *detection technologies rely on target nucleic acids being sufficiently large or abundant to be detected, and minor molecular differences in individual molecules may be beyond the limits of detection. We now present an approach for RNA detection combining the best from PRINS and FISH. Initially, an "inversed" PRINS reaction is performed in which a hybridization probe (that can be ligated to form a closed circle) is used as template and the target nucleic acid acts as primer (the opposite of the traditional PRINS approach). The subsequent DNA polymerization results in the tagging of sites of DNA synthesis with tandem repeat copies of the circular probe. This firmly localized repeated sequence can then easily be detected by FISH. The whole reaction can readily be multiplexed through the application of a cocktail of probes and subsequent visualization of the individual PRINS products with color coded identifier oligonucleotides.

Such an approach was recently presented for the analysis of DNA molecules in situ [13], combining Padlock Probes for DNA detection at single nucleotide resolution, with target primed rolling circle DNA synthesis, resulting in an amplification powerful enough to allow single molecule detection. ISH with circle probes and target primed rolling circle requires both hybridization of the probe and the presence of a 3'-end to prime the rolling circle reaction and thus is unlikely to give any signals if the probe attaches non-specifically to the target specimen. Furthermore, only part of the circle probe hybridizes to the target and by using different sequences in the non-binding part the individual probes can easily be identified in multiplex assays.

We now report a new design according to this concept, in which circular hybridization probes detect non-polyadenylated RNA molecules, initiating a rolling circle PRINS reaction from the natural 3'-end of the target RNA molecule hybridized to the probe. For this, we developed a new type of hybridization probe, a "Turtle Probe", and made modifications to previous protocols.

## Results and discussion

### Optimization and generation of new probe format

**Figure 1:**
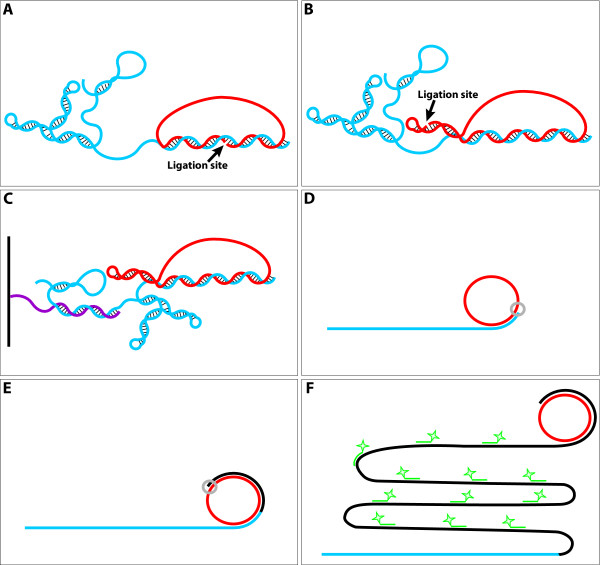
**Probe designs and methods for RNA detection**. (A) A Padlock Probe contains two ends that are brought into close proximity through hybridization to a target RNA molecule (blue) after which they can be joined by a DNA ligase (the ligation site is indicated by an arrow). The probe also contains an intervening segment which does not hybridize to the target molecule, but completes the circle. This intervening segment can be rather freely designed in terms of length and sequence and is used as the probe identifier. Thus probes may be equipped with individual intervening segments for unique identification in multiplexed experiments. (B) A Turtle Probe consists of a target recognizing element and the identifier joined by a hairpin structure bringing the probe ends into close proximity on an internal ligation template (the ligation site is indicated with by arrow). (C) Illustration of the solid support setup where the capture oligonucleotide (purple) is covalently attached to the glass, the target RNA (blue) is hybridized to the capture oligonucleotide and the Turtle Probe (red) is hybridized to the 3'-end of the target RNA. (D-F) The target RNA (blue) provides a free 3'-end for the rolling circle reaction employing a ligated circle probe (red) (could be a Padlock- or Turtle Probe) as template for the localized DNA synthesis (grey polymerase forms black DNA). The rolling circle product, extending from the 3'-end of the target RNA, is then visualized with labeled oligonucleotide probes (green) recognizing the copies of the identifier element produced in the rolling circle reaction.

Rolling circle based assays have previously been published for in situ detection of DNA, both with the addition of an external primer [17] and in a target primed assay [13]. Initially, we essentially converted the target primed DNA detection with Padlock Probes to RNA detection. Padlock Probes [18] are linear probes that are turned into closed circles when the 3'- and 5'-ends are brought into proximity by hybridization to a matching sequence, and the resulting nick is closed by a DNA ligase (Figure 1A). Although Padlock Probes can be ligated on RNA templates, this results in a reduced yield of closed circles, compared to ligation on DNA templates [19]. In histological preparations, this efficiency problem may be further aggravated by degradation (e.g. depurination) and modification (e.g. mono-methylol groups added to the bases of RNA in the common format of formalin fixed paraffin embedded (FFPE) tissue sections) of nucleic acids [20]. The performance of Padlock Probes for the detection of RNA molecules *in situ *was therefore *a priori *questionable – in particular in FFPE formats. In agreement with this, our initial results employing Padlock Probes for rolling circle detection of RNA showed low sensitivity and variation with the specimen.

In parallel with the optimization of the reaction procedures, we therefore supplemented the Padlock format with circle probes not requiring target templated ligation. One way of obtaining such probes would be by using preformed circles. Preformed circles were known before Padlock Probes, and have been employed for *in situ *detection of DNA [21]. However, the performance of the probes in that *in situ *study was not impressive compared to the performance of Padlock Probes *in situ *[13], and since the circles are formed on external linear templates, there is always the risk that some template molecules are carried over into the *in situ *reaction, where they could initiate rolling circle replication of probes not hybridized to the proper target. Since the amplification from the rolling circle reaction is sufficient for single molecule detection, any template molecule carried over would be a potential source of false signals. In practice, perfect removal of template molecules is time and labor consuming – if at all possible – since it requires e.g. gelbased purification and it would, therefore, be preferable to leave out this template. To this end we designed what we call Turtle Probes (Figure 1B). These have the desirable features of templating their own formation into closed circles and of only generating signals upon hybridization to and priming from the hybridization target. For *in situ *detection Turtle Probes have the added potential advantage over preformed circles of being closed by ligation after target hybridization, which may make them better able to wrap around the target RNA with its potential modifications and cross-links to other bio-molecules in the preparation.

While target primed detection has its advantage in securing target localization, it is also limited by the need for a properly located 3'-end in the target molecule. A collaborative study with the laboratory of Ulf Landegren on how far from the 3'-end a Padlock Probe could be positioned on a DNA template *in situ *in ethanol fixed cell lines and still result in signal showed that while signals could still be obtained with a probe positioned 134 nucleotides from the 3'-end, this position gave less signals than a position closer to the end of the target molecule [13]. Although similar tests have not been performed on FFPE tissue with RNA as target instead of DNA, the effect of the distance from the 3'-end will most likely be even more marked here. In the case of RNA, the matter is further complicated by the tendency of RNA to form secondary structures, and we have unpublished experimental indications on pure RNA in solution that the recession of the 3'-end stops when the first region of double stranded RNA is reached.

**Figure 2:**
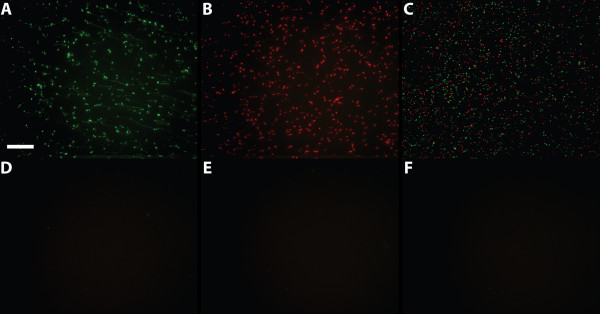
**Detection of RNA on solid support**. Solid support detection of *in vitro *transcribed RNA with Turtle Probes. (A) Detection of the HPV16E6a RNA with the probe TP-polyA-id33. (B) Detection of the SSA4-3'UTR RNA fragment with the probe TP-SSA4end-id16. (C) Combined detection of the HPV16E6a RNA and the SSA4-3'UTR RNA fragment with the probes TP-polyA-id33 (green) and TP-SSA4end-id16 (red). The two probes were co-hybridized, co-amplified, and co-detected with a mixture of Lin16 (red identifier) and Lin33 (green identifier). (D-F) Negative controls as (A) save for the following: (D) No capture oligonucleotide present; (E) No RNA was added; (F) The RNA added lacked the artificial polyA tail (HPV16E6noPA RNA) but otherwise had the same sequence as the HPV16E6a RNA used in (A). A 63× objective was used and scale bar is 50 μm.

To test the Turtle Probes in a controllable environment, they were first applied in a solid support setup, using *in vitro *transcribed RNA immobilized on a solid support through a capture oligonucleotide. After hybridization of the RNA to the capture oligonucleotide, the Turtle Probe was hybridized to the RNA (Figure 1C). The Turtle Probe was then ligated and rolling circle DNA synthesis was performed (Figure 1D–F). We tested two different Turtle Probes (TP-PolyA-id33 and TP-SSA4end-id16) with two different *in vitro *transcribed RNAs, one RNA (HPV16E6a) having an artificial polyA tail to imitate a polyadenylated RNA (Figure 2A) and the other RNA (SSA4-3'UTR fragment) having a sequence containing all four nucleotides in the 3'-end, to imitate a non-polyadenylated RNA (Figure 2B). Both Turtle Probes worked well and due to the different backbones they could easily be detected in a multiplexed format by simply adding both probes in the hybridization mixture and visualizing each rolling circle product in its specific color (Figure 2C). All the negative controls were negative (Figure 2D–G). As LNA probes have improved hybridization characteristics on RNA with its tendency to fold into secondary structures, we also tested a Turtle Probe with four LNA bases in the footprint, but this approach produced no signals (data not shown). This is probably due to a reduced polymerase activity on an LNA containing template, and we were told by the provider (Exiqon) to expect a 30–40% decrease in the activity of enzymatic reactions when using an LNA containing template. Though this had not been tested for rolling circle applications. We mainly used the solid support as test setup. However, besides providing a highly controllable environment, it also illustrates the possibilities for multiplexing Turtle Probes in e.g. chip analysis.

### *In situ *detection with Turtle Probes

**Figure 3:**
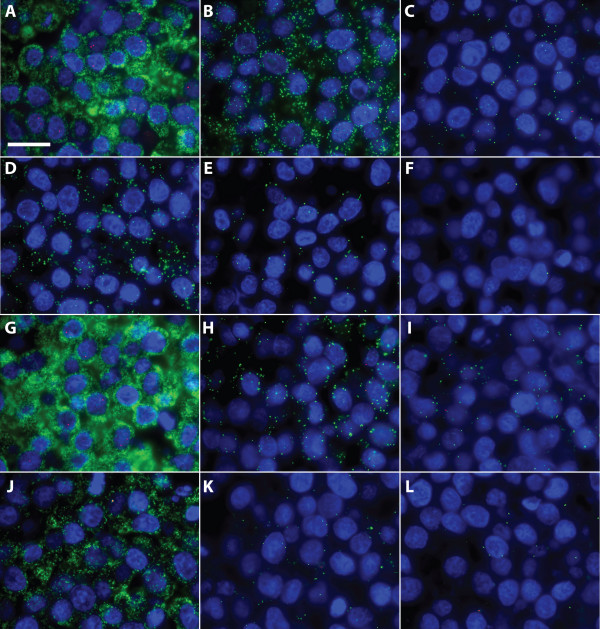
**Comparison of Turtle and Padlock Probes in FFPE cells**. Representative results of RNA detection with both Turtle and Padlock Probes in cells treated similarly to pathological routine specimens (fixed in formalin and embedded in paraffin). The counterstain is DAPI producing blue cell nuclei. A-F) Detection of 5S rRNA (green signals) and hTR (red signals) in FFPE Hela cells. A-C) RNA detection using Turtle Probes targeting 5S rRNA and hTR on FFPE Hela cells. A) Using a final Turtle Probe concentration of 100 nM. B) Using a final Turtle Probe concentration of 10 nM. C) Using a final Turtle Probe concentration of 1 nM. D-F) RNA detection using Padlock Probes targeting 5S rRNA and hTR. D) Using a final Padlock Probe concentration of 100 nM. E) Using a final Padlock Probe concentration of 10 nM. F) Using a final Padlock Probe concentration of 1 nM. hTR was only detected consistently with the highest concentration of the Turtle Probe. G-L) Detection of 28S rRNA (green signals) and hTR (red signals) in FFPE Hela cells. G-I) RNA detection using Turtle Probes targeting 28S rRNA and hTR on FFPE Hela cells. G) Using a final Turtle Probe concentration of 100 nM. H) Using a final Turtle Probe concentration of 10 nM. I) Using a final Turtle Probe concentration of 1 nM. J-L) RNA detection using Padlock Probes targeting 5S rRNA and hTR. J) Using a final Padlock Probe concentration of 100 nM. K) Using a final Padlock Probe concentration of 10 nM. L) Using a final Padlock Probe concentration of 1 nM. A 100× objective was used and scale bar is 50 μm.

For the *in situ *detection of RNA, we initially created Turtle Probes targeting the 5S and 28S ribosomal RNAs (rRNAs) and against polyadenylated RNA these were then tested in different formats [see Additional file 1]. Since the Turtle Probes worked well, we changed format to FFPE material and further modified the previous protocols, most notably by introducing pre-heating of the specimen, to reverse some of the RNA modifications introduced during the preparation of the FFPE material [20], and by combining the use of heating and carrier RNA to open potential secondary structures in the RNA. To compare the two probe types with this optimized protocol we treated Hela cells in a similar way to routine pathological tissue specimens (fixing a cell pellet in formalin and then embedding it in paraffin) and then applied Turtle Probes and Padlock Probes targeting the 5S rRNA, 28S rRNA, and hTR in a limiting dilution series to see how little probe could be used. Our expectation was that if the Turtle Probes were converted to closed circles with a higher efficiency than the corresponding Padlock Probes, the same number of signals should be obtained with less probe (less saturation of targets). Since the ligation efficiency of the Padlock Probes would be expected to vary with the target, we expected that the advantage of using Turtle Probes would show the same variation. Indeed, what we saw was an obvious but variable gain from using the Turtle Probes. For the two abundant targets, 5S rRNA and 28S rRNA, we saw more signals with the Turtle Probes at equimolar amounts of probe, and approximately the same number of signals with an order of magnitude less probe. The less abundant target, hTR, is in practice only detected with the Turtle Probe (Figure 3).

Next we tested the two types of probes on FFPE tissue material. Here we initially chose the Epstein-Barr Virus (EBV) encoded RNA, EBER1 (Epstein-Barr early RNA1), as target, since EBER1, besides being non-polyadenylated and present in high amounts in EBV positive tumors such as Hodgkin's lymphoma, also provides an internal negative control with EBER1 primarily located in the nuclei of the neoplastic Reed-Sternberg cells in this setting [22]. As a second target, we selected the RNA template for human telomerase (hTR) since, in addition to being non-polyadenylated and much less abundant than EBER1, it is also expected to be found in a subset of cells (i.e. in all Reed-Sternberg cells and less abundantly in some of the non-malignant lymphoid bystander cells) [23]. Furthermore, hTR should give rise to a few discrete signals in the nuclei, reflecting accumulation in Cajal bodies [17,24].

**Figure 4:**
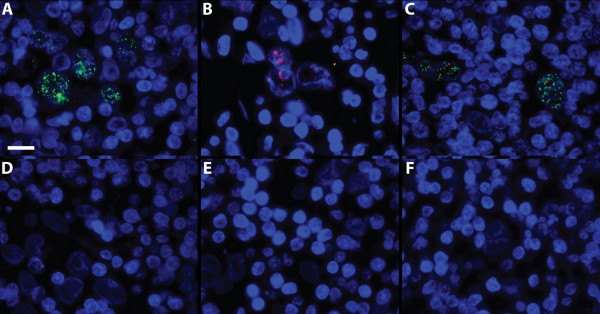
**Detection of RNA in FFPE tissue**. Representative results of RNA detection in FFPE human tissue from an anonymous patient with Epstein-Barr Virus (EBV)-associated Hodgkin's lymphoma. EBER1 RNA should appear in the neoplastic Reed-Sternberg cells and not in the surrounding lymphocytes. The counterstain is DAPI, producing blue cell nuclei. (A) Detection of EBV-encoded early RNA (EBER)1 with a Turtle Probe (TP-EBER1-id33). (B) Detection of the same target on the same material with a Padlock Probe (PP-EBER1-id16). (C) Combined detection of EBER1 and hTR with the probes TP-EBER1-id33 (green) and TPhTR-id16 (red). The two probes were co-hybridized, co-amplified, and co-detected with a mixture of Lin16 (red identifier) and Lin33 (green identifier). (D-F) Negative controls identical to (B), save for the following: (D) pretreatment with RNase, (E) exclusion of the Turtle Probe, or (F) replacement of the correct probe with a non-complementary probe (the EBV1-id16 probe, which does not recognize EBER1, but rather the BamHI repeat of EBV). A 63× objective was used and scale bar is 50 μm.

A representative result employing a Turtle Probe and the optimized protocol for EBER1 in FFPE tissue is shown in Figure 4A, and the representative result of a parallel detection with a Padlock Probe in Figure 4B. As is apparent in the illustrations, both probe formats allowed the detection of EBER1 in the neoplastic Reed Sternberg cells of EBV-associated Hodgkins lymphoma. However, stronger and more abundant signals were obtained with the Turtle Probe. The modified protocol also enabled the detection with a Turtle Probe of the housekeeping RNA hTR. This detection was done in a multiplexed reaction in which probes for EBER1 and hTR were co-hybridized, co-amplified and co-visualized (Figure 4C), showing that multiple Turtle Probes can be hybridized and rolled in parallel and analyzed individually *in situ*, if the rolling circle products are visualized with identifier probes that are unique to each Turtle Probe. For all reactions, negative controls were negative (Figure 4D–F).

## Conclusion

The present paper represents to our knowledge the first publication of RNA detection *in situ *with oligonucleotide probes and target primed rolling circle PRINS. This approach appears promising because of the high discriminatory power of the short oligonucleotide probes and the impressive amplification power of the rolling circle reaction, providing excellent signal to noise ratios in combination with exact target localization due to the target primed reaction. Furthermore, the procedure may be multiplexed to a significant extent, color-coding a number of probes reacted in parallel for combinatorial labeling. As illustrated, the approach works also when challenged with the most technically difficult preparation format, that of FFPE routine samples from the pathology archives, opening up these collections to sensitive and specific RNA analysis, something that has been possible only to a limited extent. Additionally, the technology should be appealing for the analysis of the rapidly expanding universe of small structural and regulatory RNA molecules (e.g. si- and miRNAs) [25,26]. Furthermore, we have just recently, while this paper was under revision, published a method using similar self-templating probes in combination with rolling circle DNA synthesis for production of long 5'-phosphorylated oligonucleotides [27].

## Methods

### *In vitro *transcription

The *in vitro *transcribed HPV16E6 RNA was made by purifying genomic DNA (gDNA) from SiHa cells using QIAamp DNA mini kit (Qiagen). The gDNA, either from yeast cells (donated by Torben H. Jensen) or from SiHa cells, was used as template in a PCR, using the Expand High Fidelity PCR System (Roche), and primers for either HPV16E6 or SSA4. The sequences of the primers were, for SiHa gDNA, senseHPV16-E6-T7promoter (GGAAGAAGCT TAATACGACT CACTATAGGG ATGCACCAAA AGAGAACTGC AAT) and antisenseHPV16-E6 (AGGGAATTCG AATGCGTTTT TTTTTTTTTT TTTTTTTTTT TTTTTTACAG CTGGGTTTCT CTACGTG), and for yeast gDNA, senseSSA4-3'UTR-T7promoter (GGAAGAAGCT TAATACGACT CACTATAGGG ATAAATACAA AGATGC) and antisense3'SSA-3'UTR (AGGGAATTCA ATTAACCCTC ACTAAAGGGT CGTGTTGTTT GGCG). Both sense primers contained the minimal promoter sequence recognized by the T7 RNA polymerase and a HindIII recognition site. The antisense primer for SiHa gDNA contained a stretch of 30 thymine nucleotides (to provide the RNA transcript with an artificial polyA tail), as well as both an EcoRI and a BsmI recognition site, whereas the antisense primer for yeast gDNA only had an EcoRI recognition site. The PCR products were purified by gel electrophoresis using the GFX™ PCR DNA and Gel Band Purification Kit (Amersham Biosciences now GE Healthcare) and inserted into the pUC18 plasmid (Fermentas) using the restriction enzymes HindIII and EcoRI, to provide the plasmids named pUC18-E6a and pUC18-SSA4-3'UTR. Both plasmids (amplified in competent XL1-blue bacteria) were digested with either BsmI (for generation of the HPV16E6a RNA), AflIII (for generation of the HPV16E6-noPA RNA), or EcoRI (for generation of the SSA4-3'UTR RNA fragment), purified by phenol-chloroform extraction and precipitated with ethanol prior to transcription. The *in vitro *transcription was performed with the T7 transcription kit (Fermentas) using the restriction digested plasmids as transcription template, and the RNA was purified by polyacrylamide gel electrophoresis (PAGE).

### Detection of RNA on solid support

#### Linking and hybridization reactions

The capture oligonucleotides, 3'AmHPV16E6 (GTCATATACC TCACGTCGCA GTAACTGTTG CCTTCCTTCC TTCCTT -Amin-3') and 3'AmSSA4 (AGGGAAAACT AAGAAATTCG ATGCTGCTAC CCTTCCTTCC TTCCTT-Amin-3'), were coupled to CodeLink activated slides (Amersham Bioscience) according to the manufactures protocol, except that the spotted area (approximately 2.5 mm^2^) was encircled with a DAKO-pen (Dako) and spotted by adding 10 μL of capture oligonucleotide mixture to the slide. The following reactions were all performed in a total reaction volume of 5 μL. Following all hybridizations or enzymatic reactions, the slides were washed in wash buffer (0.1 M Tris-HCL (pH 7.5, at 25°C), 0.15 M NaCl, 0.05% Tween-20) at room temperature (RT). The duration of the wash was 3 min after the hybridization steps and 1 min after the enzymatic steps. *In vitro *transcribed RNA (0.25 μM RNA was used in a total volume of 5 μL hybridization mixture, corresponding to final amounts of approximately 21 ng SSA4 RNA, 37 ng HPV16E6-noPA and 41 ng HPV16E6a, respectively) was hybridized to the capture oligonucleotide in a mixture containing: 0.5 M NaCl, 10 mM Tris-HCl (pH 7.5, at 25°C), 1 mM EDTA, 0.01% SDS, 10 mM DTT and 1 u/μL Ribolock RNase Inhibitor (Fermentas). The hybridization reaction took place in a humidity chamber over night (16 hours) at 37°C. The Turtle Probes, TP-SSA4end-id16 (p-GTCGATCCCC TCAATGCTGC TGCTGTACTA CAATTCAATT AACCCTCACT AAAGGGTCGT GGGATCGACT CGGAATAACC GA) and TP-PolyA-id33 (p-CATTCTCCCC TCAATGCACA TGTTTGGCTC CTTTTTTTTT TTTTTTTTTT TTTTTTTTTT TGGAGAATGC GAGAATAACT CG) were hybridized in a final concentration of 0.1 μM in a total volume of 5 μL, under the same conditions as the RNA, though only for 30 min at 37°C in the humidity chamber.

#### Enzymatic reactions

Ligation was performed with 0.1 u/μL T4 DNA Ligase (Fermentas) in 1× T4 DNA ligation buffer (supplied with the T4 DNA ligase) supplemented with 1 u/μL Ribolock RNase Inhibitor (Fermentas) and 0.2 μg/μL BSA in a humidity chamber for 30 min at 37°C. The rolling circle was performed with 1 u/μL phi29 DNA polymerase (Fermentas) in 1× phi29 buffer (supplied with the phi29 DNA polymerase) supplemented with 1 u/μL Ribolock RNase Inhibitor (Fermentas), 0.2 μg/μL BSA and 0.25 mM dNTP in a humidity chamber for 30 min at 37°C.

#### Hybridization of detection probes

The fluorescent probes, Lin16 (5'-Rhodamine-CCTCAATGCT GCTGCTGTAC TAC) and Lin33 (5'-FITC-CCTCAATGCA CATGTTTGGC TCC) were hybridized to the rolling circle product in a concentration of 0.2 μM of each in a total volume of 5 μL, in a mixture containing 0.5 M NaCl, 10 mM Tris-HCl (pH 7.5, at 25°C), 1 mM EDTA, 0.01% SDS, 10 mM DTT and 1 u/μL Ribolock RNase Inhibitor (Fermentas) in a humidity chamber for 30 min at 37°C. The slides were washed in wash buffer 2 × 3 min, dehydrated in 99% EtOH, drained for excess ethanol, air dried, and mounted with VectaShield (Vector Laboratories).

### Detection of RNA in FFPE cell line

#### Pretreatment of FFPE cell line

Hela cells were grown to confluenc, spun down and the cell pellet was fixed in 10% buffered formalin and embedded in paraffin. Sections (4 μm thick) were cut from the FFPE Hela cell pellet block using a standard microtome, placed on SuperFrost^®^Plus glass slides (Menzler-gläser) and incubated for 45 min at 65°C. The sections were deparaffinized in xylene for 2 × 10 min. The xylene was extracted in 99.9% (vol/vol) ethanol for 4 × 2 min, in 85% (vol/vol) ethanol for 2 × 2 min, and in 99.9% (vol/vol) ethanol for 1 × 2 min, the slides were drained for excess ethanol and air dried. The sections were submerged in 0.6 u/μL pepsin (solid units, Sigma) in 0.1 M HCl and incubated at 37°C for 8 min. Pepsin concentration and incubation time may need to be optimized according to the length of fixation, the type of tissue, and the type of sample. Pepsin treatment was stopped by submerging slides in wash buffer for 2 min (wash buffer: 0.1 M Tris-HCL (pH 7.5, at 25°C), 0.15 M NaCl, 0.05% Tween-20), after which the slides were dehydrated through an ethanol series (70%, 85%, 99.9% (vol/vol)), drained for excess ethanol, and air dried.

#### Probe hybridization

The tissue sections were encircled with a DAKO-pen (Dako), and a hybridization mixture containing 20% formamide, 2× SSC, 5% glycerol, 1 μg/μL carrier RNA (Qiagen) and 100 nM, 10 nM or 1 nM Turtle Probe, TP-5S rRNA (p-GTCGATCCCC TCAATGCACA TGTTTGGCTC CAAAGCCTAC AGCACCCGGT ATTCCCAGGC GGGATCGACT CGGAATAACC GA), TP-28S rRNA (p-GTCGATCCCC TCAATGCACA TGTTTGGCTC CGACAAACCC TTGTGTCGAG GGCTGACTTT CGGATCGACT CGGAATAACC GA), TP-hTR (p-GTCGATCCCC TCAATGCTGC TGCTGTACTA CGCATGTGTG AGCCGAGTCC TGGGTGCACG TCCCACAGCT CGGATCGACT CGGAATAACC GA), or Padlock Probe, PP-5S rRNA (p-CGGTATTCCC AGGCGTTTAT TTCCTCAATG CACATGTTTG GCTCCTAGTG ATTTACTTGG ATGTCTAAAG CCTACAGCAC C), PP-28S rRNA (p-CGAGGGCTGA CTTTCTTTAT TTCCTCAATG CACATGTTTG GCTCCTAGTG ATTTACTTGG ATGTCTGACA AACCCTTGTG T), PP-hTR (p-TGGGTGCACG TCCCACAGCT CTTTATTTCC TCAATGCTGC TGCTGTACTA CTAGTGATTT ACTTGGATGT CTGCATGTGT GAGCCGAGTC C) was added to the slide. The hybridization was performed under a coverslip sealed with heat-resistant glue, and for optimal temperature control the slide hybridization was performed using a Twin Tower PTC-200 (MJ Research, Waltham, MA, USA) programmed as follows: Heat to 95°C for 2 min, RAMP 0.5°C/s to 37°C and 37°C for 30 min. The slides may be left at 37°C as long as overnight, provided they do not dry out during the extended incubation. To remove unbound probe, slides were washed separately for 5 min in 2× SSC + 0.05% Tween-20 preheated to 37°C, and washed for 5 min in wash buffer preheated to 37°C. (Slides were washed separately since single molecule sensitivity carries the inherent risk of cross contamination).

#### Enzymatic reactions

Ligation of Turtle Probes was performed in 1× T4 DNA ligation buffer (supplied with the T4 DNA ligase), 0.2 μg/μL BSA, and 0.1u/μL T4 DNA ligase (Fermentas). Ligation of Padlock Probes was performed in the buffer described in Nilsson et al. (2000). Ligation reactions, were incubated under coverslip at 37°C for 30 min in a humidity chamber, washed in wash buffer for 3 min at room temperature, dehydrated and air-dried. The rolling circle was performed in 1× Phi29 buffer (supplied with the Phi29 DNA polymerase), 0.2 μg/μL BSA, 0.25 mM dNTP, 5% glycerol, and 1 u/μL Phi29 DNA polymerase (Fermentas) under a coverslip at 37°C for 45 min in a humidity chamber. Following the rolling circle, slides were washed in wash buffer for 3 min at RT, dehydrated and air-dried.

#### Hybridization of detection probes

The rolling circle products were detected by hybridizing the fluorescently labeled identifier probes lin16 and lin33 (0.2 μM of each) in a mixture containing 20% formamide, 2× SSC, and 5% glycerol under coverslip for 30 min at 37°C in a humidity chamber (may be left over night at 37°C). The slides were washed in wash buffer 2 × 5 min at room temperature, dehydrated, and mounted with VectaShield with DAPI (Vector Laboratories).

### Detection of RNA in FFPE tissue

All steps were performed as for detection of RNA in FFPE cell line except that the Turtle Probe, TP-hTR (p-GTCGATCCCC TCAATGCTGC TGCTGTACTA CGCATGTGTG AGCCGAGTCC TGGGTGCACG TCCCACAGCT CGGATCGACT CGGAATAACC GA), TP-EBER1 (p-GTCGATCCCC TCAATGCACA TGTTTGGCTC CAAAACATGC GGACCACCAG CTGGTACTTG ACCGGATCGA CTCGGAATAA CCGA) or Padlock Probe, PP-EBER1 (p-CAGCTGGTAC TTGACCCCTC AATGCTGCTG CTGTACTACT AGTGATTTAC TTAAAACATG CGGACCAC) was used in a final concentration of 100 nM.

### Image analysis

Both solid support- and tissue-slides were analyzed in a Leica epifluorescence microscope and images recorded with a SenSys CCD-camera operated by the SmartCapture 2 version 2.0 image analysis from digitalscientific (Cambridge, UK).

## Authors' contributions

MS contributed to the conception of the method, probe design, performed all experiments in the laboratory, and drafted the manuscript. JSL participated in method development, probe design, data analysis and revision of the manuscript. MBZ participated in method development and revision of the manuscript. SHD contributed with data analysis and language/grammatical revision of the manuscript. JK initiated the project and contributed with data analysis and revision of the manuscript. All authors read and approved the final manuscript.

## Supplementary Material

Additional file 1 Detection of RNA in non-FFPE formats. Detection of RNA with Turtle Probes in ethanol fixed cell line and formaldehyde fixed C. elegans. Click here for file
