# Supplementary material for: In situ detection of non-polyadenylated RNA molecules using Turtle Probes and target primed rolling circle PRINS
Source: BMC Biotechnol. 2007 Oct 18;7:69. doi: 10.1186/1472-6750-7-69 (PMC2203993; doi:10.1186/1472-6750-7-69)
Supplement: Additional file 1 — Detection of RNA in non-FFPE formats. Detection of RNA with Turtle Probes in ethanol fixed cell line and formaldehyde fixed C. elegans. [file 1472-6750-7-69-S1.pdf]

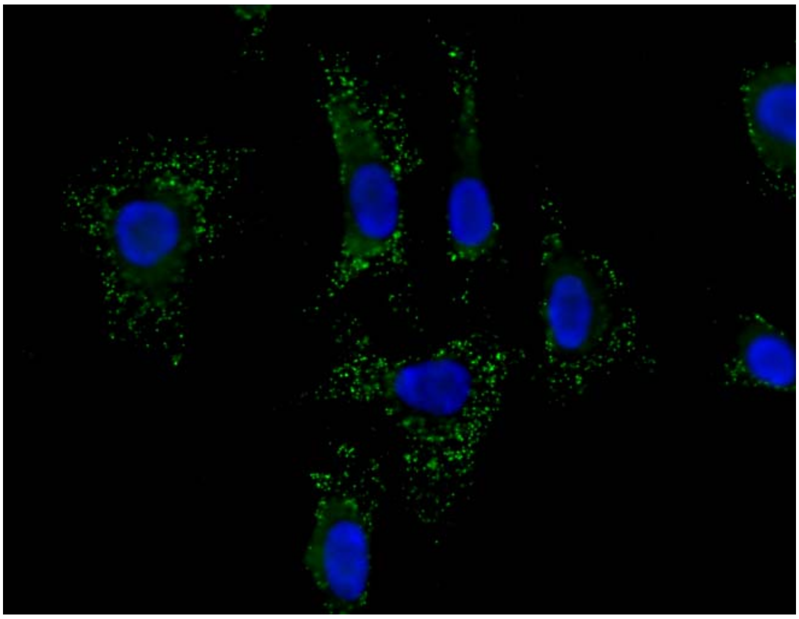

Detection of 28S rRNA in EtOH fixed cells.
Cells were grown on SuperFrost®Plus glass slides (Menzler-gläser), washed in 1xPBS for 2 min at 4°C and fixed/permabilized in EtOH over night at 4°C. The slides were air dried, hybridization mixture containing 20% formamide, 2x SSC, 5% glycerol, 1 µg/µL carrier RNA (Qiagen) and 100 nM Turtle Probe was added and the slides incubated for 30 min at 37°C. The subsequent reactions were performed as described for “detection of RNA in FFPE tissue”.

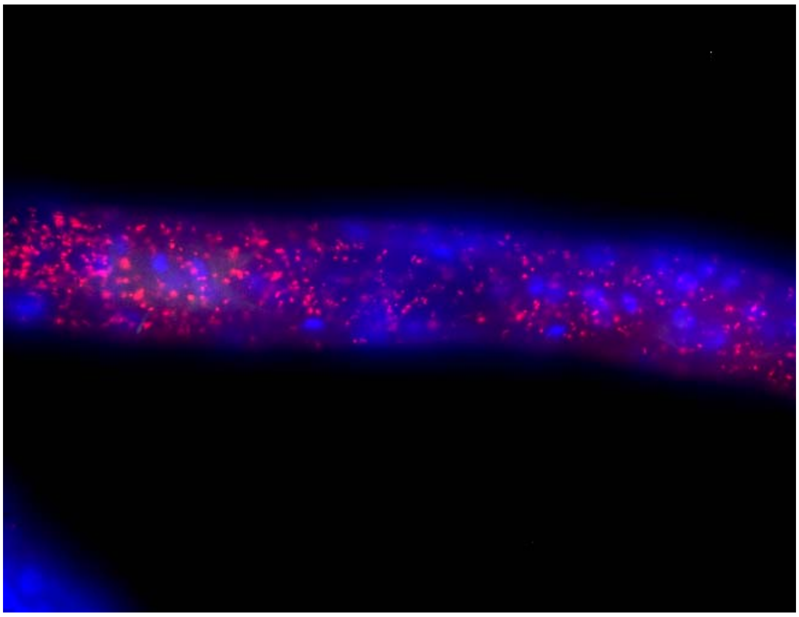

Detection of polyadenylated RNA in formaldehyde fixed *C. elegans*.
Methods for handling and culturing *C. elegans* were essentially as described previously by Benner S. in Genetics. 1974 May; 77(1):71-94 and fixation and permabilization as described by Gary Ruvkun and Michael Finney in the protocol: Antibody Staining of Formaldehyde-fixed Worms. Except that the worms after oxidization were fixed in formaldehyde, dehydrated in an EtOH/PBS series and stored in 99% EtOH at 20°C. Sample of worms were added to SuperFrost®Plus glass slides (Menzler-gläser) and air dried. The worms were digested with 0.3 u/µL pepsin (solid units, Sigma) in 0.1M HCl and incubated at 37°C for 4 min. Pepsin treatment was stopped by incubation in wash buffer for 2 min (wash buffer: 0.1 M Tris-HCL (pH 7.5, at 25°C), 0.15 M NaCl, 0.05% Tween-20), after which the slides were dehydrated through an ethanol series (70%, 85%, 99.9% (vol/vol)), drained for excess ethanol, and air dried. The subsequent reactions, hybridizations and enzymatic reactions, were performed essentially as described for “detection of RNA in FFPE tissue” with the exception of carrier RNA was not added.
